# Non-puerperal uterine inversion secondary to prolapsed uterine fibroid in a 21-year-old lady with favorable outcome: a case report

**DOI:** 10.1097/RC9.0000000000001109

**Published:** 2026-10-14

**Authors:** Nzometia C. Yimlefack, Forchap N. Milton, Ashu J. Nchung, Wamba A. Gabrielle, Sow Aissatou, Nana D. Theophille

**Affiliations:** a Department of Obstetrics/Gynaecology, Faculty of Health Sciences, University of Buea, Buea, Cameroon; b Department of Obstetrics and Gynaecology, Buea Regional Hospital, Buea, Cameroon; c Department of Obstetrics & Gynaecology, Douala General Hospital, Douala, Cameroon

**Keywords:** Cameroon, non-puerperal, prolapsed uterine fibroids, uterine inversion

## Abstract

**Introduction and importance:**

Non-puerperal uterine inversion (NPUI) is exceedingly rare in younger women. In a resource-poor setting, it poses both diagnostic and therapeutic challenges.

**Case presentation:**

We present a 21-year-old G0P0, sexually active woman who presented with a 1-week history of vaginal bleeding complicated by hypovolemic shock in the context of a prolapsed 8 cm myoma beyond the introitus. Her hemoglobin level on admission was 6 g/dL. Following an initial transfusion of 2 units of whole blood, successful twisting and untwisting of the protruding myoma on its pedicle (vaginal myomectomy/bistournage) was performed. Following tumor excision (vaginal myomectomy), repeat pelvic examination revealed a persistent mass protruding through the dilated cervical os, and an inability to define the borders of the cervix. Attempts at manual transvaginal reduction of the inverted uterus failed and triggered brisk vaginal hemorrhage. Failure to reposition the uterine fundus to its normal anatomical position led to a laparotomy with uterine repositioning. A horizontal incision on the anterior uterine wall was made, with successful repositioning of the uterine fundus. Her postoperative recovery was uneventful, and she was dischared on the fifth day and was followed up on an outpatient basis, with return of normal menstrual flow a month later.

**Clinical discussion:**

This case highlights the unusually early onset of symptomatic fibroids in young Black women presenting with NPUI in resource-limited settings where immediate diagnostic facilities are unavailable. An anterior transverse uterine incision, performed to reposition the uterine fundus in the context of NPUI, successfully repositioned the uterus.

**Conclusion:**

The management of NPUI due to fibroids in young women may be associated with an increased chance of surgical intervention. In a resource-poor setting, the bistournage technique may upstage NPUI, as the diagnosis is made after the resection of the prolapsed cervical fibroid. Our case presents a very rare case of a fibroid in a 21-year-old and demonstrates that an anterior transverse uterine incision of approximately 2 cm also gives the surgeon access to reposition the fundus of the uterus.

## Introduction

Uterine inversion is a condition in which the fundus of the uterus turns inside out and the uterus prolapses through the cervix; it is classified into two types: puerperal or obstetric and non-puerperal or gynecological uterine inversion^[^1,2^]^. The non-puerperal uterine inversion (NPUI) is mostly related to benign or malignant tumors associated with the uterine corpus, presenting mostly as chronic cases, although Das *et al* reported that 8.6% of NPUI cases were of sudden onset^[^2,3^]^.

HIGHLIGHTS

- Non-puerperal uterine inversion (NPUI) in an adolescent caused by a probable fibroid.
- NPUI presents significant diagnostic challenges in resource-limited settings.
- A horizontal anterior uterine incision resulted in successful uterine repositioning.

NPUI is rare; its incidence is unknown, and the mean age at presentation is 46.3 years. Leiomyomas are the most common cause of NPUI^[^2^]^.

The mechanism of NPUI is not entirely clear. If the myometrium becomes distended due to a tumor within the cavity, it becomes irritable and initiates expulsive contractions, which can dilate the cervix and assist in the expulsion of the tumor, thereby placing traction on its fundal attachment. The weight of the tumor, manual traction on the tumor, or increased intra-abdominal pressure due to coughing, straining, and sneezing may also contribute to NPUI^[^2,4^]^. This case report has been prepared in line with the SCARE checklist, with an attached CARE-SCARE clinical timetable (Table 1)^[^5^]^.

**Table 1:** CARE/SCARE timeline table with dates.

Time/interval	Date	Clinical event	Category (CARE/SCARE item)
1 Week	26/03/2026– 01/04/2026	Onset of vaginal bleeding and protruding vaginal mass	History of illness
Day 0	02/04/2026	Presentation in our hospital	Chief Complaint/presenting concern
Day 0	02/04/2026	Blood transfusions, vaginal myomectomy, open laparotomy	Resuscitation, surgery
Day 5	07/04/2026	Discharge	Good progress following surgery
Day 12	14/04/2026	Outpatient follow up	Good progress following surgery
Day 35	27/05/2026	Return of menses, lasting 4 days	Ruling out the possibility of endometrial scarring, preservation of uterine factor of female fertility
Day 65	24/06/2026	Onset of menses, lasting 5 days	Ruling out the possibility of endometrial scarring, preservation of uterine factor of female fertility

## Case presentation

We present the case of a 21-year-old nulligravid, sexually active woman (blood group O positive) who presented to the emergency department of Buea Regional Hospital with a 1-week history of severe vaginal bleeding accompanied by severe fatigue and blurred vision. She denied fever, pelvic pressure, or abdominal pain. Upon initial examination, the patient was lethargic and acutely ill-appearing, with severe conjunctival pallor. Physical signs of hypovolemic shock were present: blood pressure was 80/65 mmHg, heart rate was 134 beats per minute, and axillary temperature was 37.1°C. Gynecological examination revealed a soft, regular mass measuring 8 cm in diameter protruding through the vaginal introitus, which bled readily upon contact. Digital vaginal examination confirmed a pedunculated mass; however, the cervix could not be clearly demarcated. Speculum examination was discontinued due to active hemorrhaging. Bimanual rectovaginal examination demonstrated an inability to palpate the uterine fundus in its anatomical position.

Laboratory workup revealed a hemoglobin level of 6.0 g/dL. Serum human chorionic gonadotropin was negative, and serological screening for human imunodefeciency virus, hepatitis B, and hepatitis C was non-reactive. The initial clinical impression was a prolapsed, pedunculated cervical leiomyoma complicated by severe acute hemorrhagic anemia (Fig. 1A).

**Figure 1.**
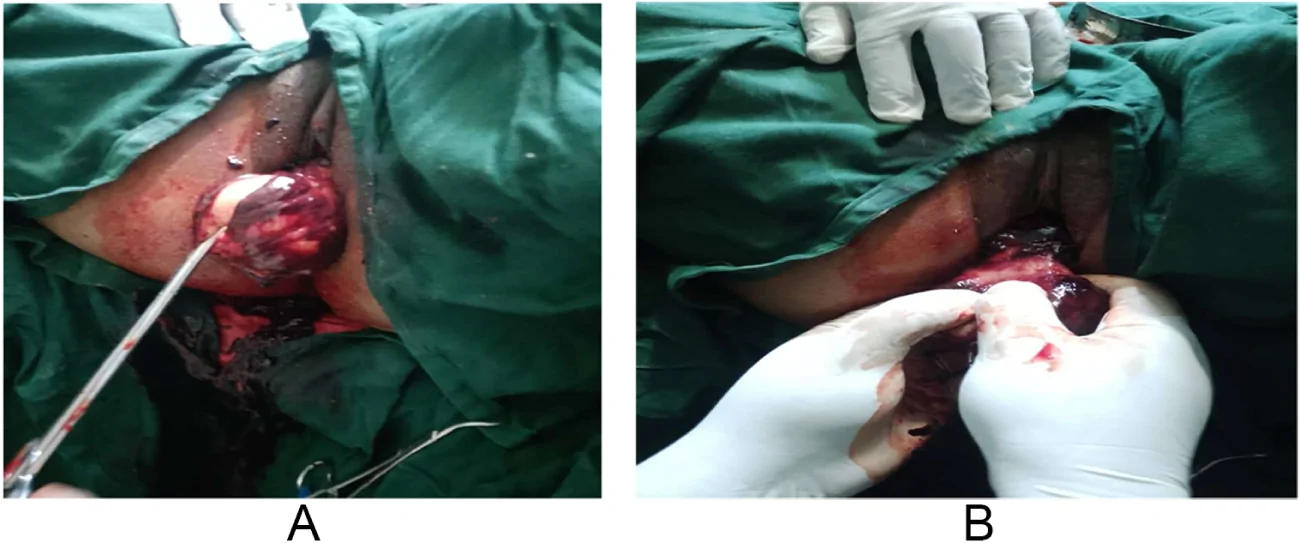
Prolapsed uterine fibroid through the introitus. (A) Prolapsed fibroid through the introitus. (B) Bistournage of the prolapsed fibroid: vaginal myomectomy.

Resuscitation was initiated immediately with intravenous fluids and 2 units of whole blood. Under general anesthesia, a vaginal myomectomy was executed via rotational traction on the tumor pedicle (bistournage; Fig. 1B). Following tumor excision, repeat pelvic examination revealed a persistent mass protruding through the dilated cervical os, along with an inability to define the cervical rim. Attempts at manual transvaginal reduction of the inverted uterus failed, triggering brisk vaginal hemorrhage.

Emergency exploratory laparotomy was performed via a Pfannenstiel incision. Intraoperative inspection confirmed a stage II NPUI: the uterine fundus was deeply invaginated, with bilateral round ligaments and adnexa pulled into the constriction ring (Fig. 2A). Traumatic lacerations were noted along the anterior uterine wall. A 2 cm anterior transverse hysterotomy was performed across an existing laceration on the anterior uterine wall. Gentle upward traction combined with manual manipulation through the hysterotomy successfully repositioned the uterine fundus into its normal anatomical orientation (Fig. 3A). The hysterotomy and anterior uterine wall defects were repaired in a single-layer using simple interrupted 2 polyglactin 910 (Vicryl) sutures (Fig. 3B). Total surgical duration was 40 minutes, and total estimated blood loss was 100 mL. Perioperative antibiotic prophylaxis consisted of intravenous ceftriaxone (2 g daily) and metronidazole 500 mg 08 hourly for 72 hours.

**Figure 2.**
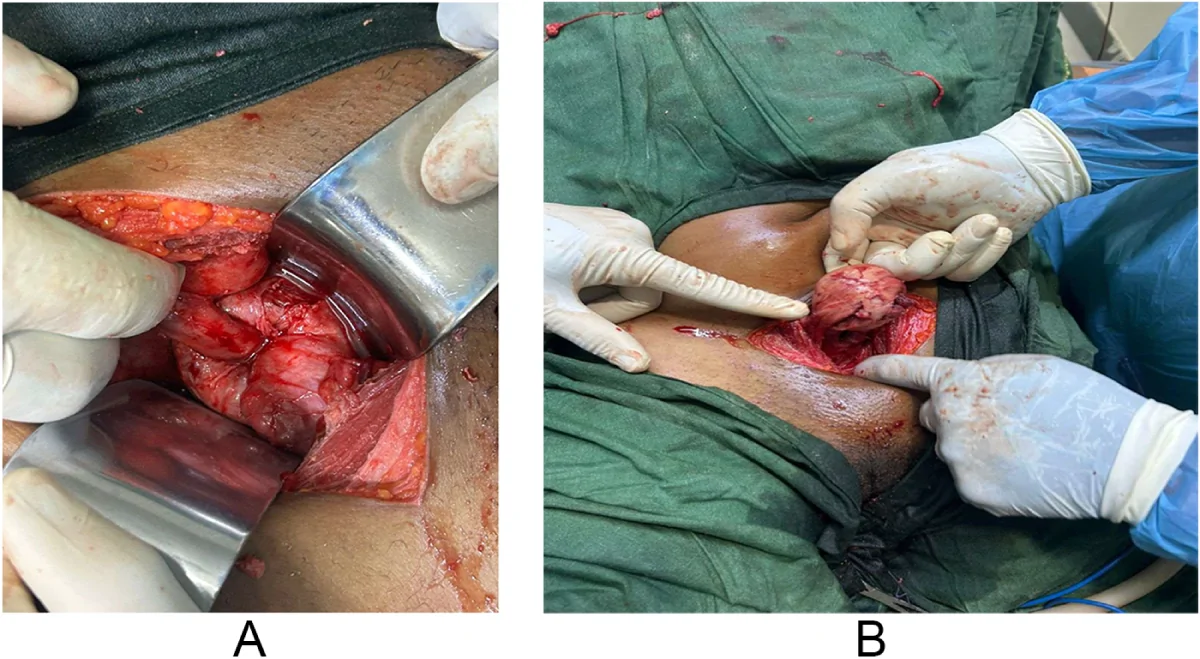
Uterine inversion. (A) Intraoperative image of nonpuerperal uterine inversion. (B) Repositioned uterus with lacerations on the anterior wall.

**Figure 3.**
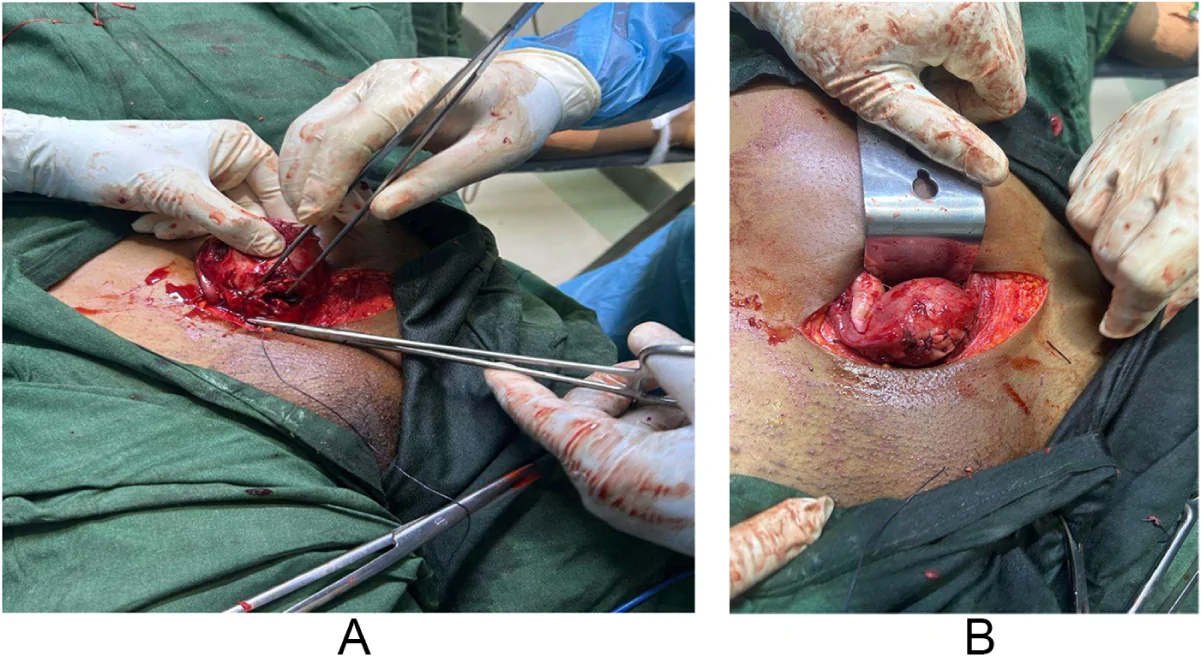
Repair of a horizontal anterior uterine incision. (A) Horizontal anterior uterine incision being repaired. (B) Repositioning the fundus of the uterus after the repair.

The excised tissue specimen was preserved in 10% formalin and released to the family alongside pathology requisition forms. However, histopathological analysis could not be completed due to financial limitations that prevented the family from submitting the specimen to the pathology laboratory. The patient’s postoperative course was uneventful. Early ambulation was initiated on postoperative day 1. Hemoglobin at discharge on postoperative day 5 was 8.1 g/dL; she was prescribed 100 mg of oral elemental iron daily for 3 months. At her 1-month outpatient follow-up consultation, she reported the complete resolution of symptoms and the return of regular menses. We explained the potential risk of uterine rupture and the likely need for an elective cesarean section should she become pregnant in the future.

## Discussion

Approximately 200–300 cases of NPUI have been reported in the global literature. Uterine leiomyomas account for roughly 80% of these cases, with the remainder primarily attributed to uterine sarcomas and other malignancies. Historically, terminology describing the underlying etiologies varied considerably across publications due to changes in diagnostic classification over several decades. Based on available historical data, carcinomas and sarcomas each accounted for 8.5%, while malignant mixed Müllerian tumors contributed 6.5% of reported cases^[^5–8,10^]^. The mean age at presentation across all causes was 46.3 years^[^2^]^. Our 21-year-old patient may be among the earliest documented cases of NPUI secondary to a probable fibroid in the medical literature. Given her young age and low baseline risk, screening for cervical malignancy was not pursued.

The most commonly known clinical presentations were foul-smelling vaginal discharge and irregular vaginal bleeding, some cases to the extent of causing anemia requiring blood transfusions^[^2,6^]^. Our patient presented with a 1-week history of vaginal bleeding and clinical signs of decompensated anemia (severe fatigue, blurred vision, tachycardia), and a hemoglobin level of 6 g/dL. This delay in presenting to the hospital explained her very low hemoglobin level.

Imaging techniques such as ultrasound and magnetic resonance imaging (MRI) play a key role in the diagnosis of NPUI. However, the diagnostic value of transvaginal ultrasound is limited, especially when a large mass protrudes into the vagina, because of physical limitations: the probe cannot be inserted into a narrowed vaginal canal. Another issue is that ultrasonic beams cannot be transmitted beyond the mass, resulting in low-quality sonographic images. MRI is the preferred diagnostic tool and can provide an accurate diagnosis before surgery^[^9^]^. In our setting, we did not have access to either an emergency ultrasound or an MRI scan, as these services are unavailable in the hospital. This made the initial diagnosis of NPUI very difficult, a difficulty compounded by her age.

NPUI is most often triggered by internal forces. These internal forces include sudden extrusion of a tumor from the uterus, thinning of the uterine wall, dilatation of the uterine cervix, tumor size, thickness of the tumor pedicle, and tumor attachment site^[^2,4^]^. Our case involved a submucosal uterine fibroid measuring about 8 cm. Its weight, coupled with cervical dilatation, likely pulled and weakened the uterine fundus. This resulted in the delivery of the myoma and uterine inversion. Intraoperatively, there was complete inversion of the uterine fundus through the fibromuscular ring of the cervix, reflecting stage II NPUI^[^2^]^.

Given her age, fertility preservation was our goal. Repositioning of the uterine fundus was unsuccessful after the vaginal myomectomy (twisting and untwisting the fibroid on its pedicle). The Spinell and Kustner techniques are the two most successful organ-preserving vaginal approaches reported in the literature for NPUI. In the Spinell procedure, an anterior longitudinal uterine incision is made after bladder dissection, whereas in the Kustner technique, the incision is made in the posterior wall^[^2,9^]^. In our case, an anterior horizontal incision was made on the uterus via laparotomy, following an existing laceration on the anterior wall, which led to successful repositioning of the uterine fundus (Figs 2B, 3A, and 3B). Given our context-specific limitation in confirming the histopathological diagnosis, we could not definitively rule out other possible causes of NPUI, such as sarcomas. The reasons for the family’s decision to discard the resected mass were not communicated to our team. In our context, such patients report financial constraints. This adds to the peculiarity of our case, given the absence of emergency imaging services, highlighting the challenges of resource-poor settings such as ours.

Reflecting on why the manual vaginal repositioning failed after the initial vaginal myomectomy, we postulate that the blind twisting and traction applied to the fibroid pedicle may have exacerbated an undetected uterine inversion. This mechanical stress explains the anterior uterine lacerations observed intraoperatively (Fig. 2B). We learned that when managing prolapsed vaginal masses in low-resource settings, we should be aware of the possibility of an underlying uterine inversion, especially in the context of excessive traction.

## Conclusion

NPUI in young women is exceedingly rare and hence poses a diagnostic and therapeutic challenge in resource-limited settings such as ours. A high index of suspicion, rapid resuscitation, and prompt surgical intervention via a fertility-preserving laparotomy may offer a favorable outcome.

## Data Availability

All data from this study are included in this published article and its supplementary files.
